# Biodegradable Poly(ε-Caprolactone) Active Films Loaded with MSU-X Mesoporous Silica for the Release of α-Tocopherol

**DOI:** 10.3390/polym12010137

**Published:** 2020-01-06

**Authors:** Cristina Mellinas, Marina Ramos, Aida Grau-Atienza, Anna Jordà, Nuria Burgos, Alfonso Jiménez, Elena Serrano, María del Carmen Garrigós

**Affiliations:** 1 Department of Analytical Chemistry, Nutrition & Food Sciences, University of Alicante, San Vicente del Raspeig, ES-03690 Alicante, Spain; cristina.mellinas@ua.es (C.M.); marina.ramos@ua.es (M.R.); annajordasanchez@gmail.com (A.J.); nuria.burgos@ua.es (N.B.); alfjimenez@ua.es (A.J.); 2 Department of Inorganic Chemistry, Molecular Nanotechnology Laboratory, University of Alicante, San Vicente del Raspeig, ES-03690 Alicante, Spain; aida.grau@ua.es

**Keywords:** mesoporous silica, α-tocopherol, biodegradable active films, Poly(ε-caprolactone), controlled release

## Abstract

In this study, new active PCL (poly(ε-caprolactone)) films containing α-tocopherol (TOC) and MSU-X mesoporous silica were prepared by melt blending. The studied additives were directly incorporated into the polymer matrix or by impregnating TOC into MSU-X silica (PCL-IMP). Thermal, optical, oxygen and water barrier properties as well as oxidation onset parameters, were studied. Films containing MSU-X and/or TOC showed a significant increase in oxidative onset temperature (OOT) and oxidative induction time (OIT), improving thermal stability against materials oxidation by the addition of mesoporous silica and TOC into the polymer matrix. In addition, the effect of MSU-X addition on the migration behaviour of α-tocopherol from active films was investigated at 40 °C using 50% (*v*/*v*) ethanol as fatty food simulant, showing PCL-IMP films the lower release content and diffusion coefficient (3.5 × 10^−15^ cm^2^ s^−1^). Moreover, radical scavenging (DPPH and ABTS) and antibacterial activity against *E. coli* and *S. aureus* were favoured by the release of α-tocopherol in the developed films. The obtained results have demonstrated the potential of the new PCL-based active formulations for TOC controlled release in antioxidant and antibacterial food packaging applications.

## 1. Introduction

Interest in the production of bio-based and biodegradable materials for food packaging applications is growing, largely motivated by the social demand to reduce the environmental impact of plastic waste caused by the non-biodegradability of the common petrochemical-based polymers [1]. Poly(ε-caprolactone) (PCL) is one of the most studied biodegradable polymers due to its potential use in biomedical (with FDA approval), pharmaceutical and packaging film applications [2,3,4]. PCL is a semicrystalline polyester typically obtained by the ring-opening polymerization reaction of the caprolactone monomer, using stannous octoate as catalyst and low molar mass alcohols as chain extenders [5]. This biopolymer presents some advantages, such as large processing capacity, biocompatibility, safety and high-water resistance due to its hydrophobic character. However, its production in large scale, especially in the packaging field, is limited by its relatively high price, low barrier properties and restricted mechanical performance due to its low melting point [6].

In order to overcome these drawbacks, some authors have suggested the combination of PCL with nano-reinforcements with the aim of improving mechanical, thermal or barrier properties, allowing the obtained materials to meet with industrial specifications [4,6,7,8]. In addition, the combination of nano-reinforced matrices with active antioxidant compounds could be considered a promising alternative for the controlled antioxidant release in active packaging films, prolonging the action time of the antioxidant packaging and improving the effect of the active substance in inhibiting food rancidity, extending shelf-life and controlling food safety [9]. A current increasing trend is the replacement of synthetic additives by the incorporation of active compounds coming from a natural origin into polymer matrices due to their lower toxicity and higher safety, providing antioxidant and/or antimicrobial properties to the active packaging systems [10,11].

Nano- or microencapsulation can be carried out using different carriers to incorporate the active additives. Dias et al. [12] demonstrated the antioxidant capacity and the controlled release of α-tocopherol incorporated to montmorillonite by using PCL as the polymer matrix. Sogut et al. [8] used chitosan and PCL-based films containing nanocellulose (2% w/w) and grape seed extract to obtain bilayer films for transferring functional polyphenolic compounds in food packaging applications. Other alternative is the incorporation or combination of the active compound with a mesoporous matrix as support material for encapsulation. Mesoporous silica particles exhibit unique features, such as high stability, biocompatibility, non-apparent toxicity, large load capacity and the possibility of including gate-like scaffoldings on the external surface [13]. In addition, these materials have been proved to be an effective method for controlling the release rate of active compounds [14,15,16,17,18]. The use of mesoporous silica particles presents a suitable approach to avoid some problems such as complex mass transport phenomena, interaction with food constituents or functional modifications due to physical and chemical food processes. These encapsulation systems also allow preserving the stability of the active additive and controlling its release rate.

Among various natural active additives, α-tocopherol (TOC) is known to be a potent antioxidant agent and films loaded with this antioxidant have been proved to be effective in retarding lipid oxidation [19,20,21]. This natural additive is authorized as food contact material by EFSA and FDA authorities [22], and it can provide antioxidant/antimicrobial properties to the food product while also stabilizing the polymer matrix during processing [12]. The incorporation of α-tocopherol into different biopolymers, in particular poly(lactic acid) (PLA) [23,24,25,26] and edible polymers such as methylcellulose [27] or chitosan [19], has been reported. Regarding mesoporous silica-based materials, Heirlings et al. [28] studied the release of α-tocopherol loaded with SBA-15 from LDPE films, whereas Gargiulo et al. [18] observed a reduction in the release rate of α-tocopherol by using amino-functionalized SBA-15. Similarly, Sun et al. [17] confirmed that the smaller pore size distribution of MCM-41 was more effective for the controlled release of α-tocopherol compared to the use of SBA-15.

To the best of our knowledge, no research study has been found dealing with the use of MSU-X silica and α-tocopherol for their incorporation and release study into biodegradable active packaging systems. Therefore, the aim of the present work is the development and characterization of new active PCL-based films containing α-tocopherol and MSU-X mesoporous silica. The characterization of the developed materials included the study of thermal, barrier, optical antioxidant, and antimicrobial properties and the evaluation of the kinetic release of α-tocopherol.

## 2. Materials and Methods

### 2.1. Materials

Tetraethoxysilane (TEOS, >99.5%) was used as silica source and ammonium fluoride (NH_4_F, >98%) was used as nucleophilic catalyst for the condensation of the silica network were provided from Sigma-Aldrich (Darmstadt, Germany). Triton X-100 (Alfa Aesar, Ward Hill, MA, USA, EEUU) was used as structure-directing agent. Commercial α-tocopherol (TOC, Sigma-Aldrich, Darmstadt, Germany) was selected as antioxidant/antimicrobial compound. Poly(ε-caprolactone) (PCL) Capa^TM^ 6800 was supplied by Perstorp (Malmö, Sweden) in granular form with mean molecular weight of 8 × 10^4^ g mol^−1^, melting point between 58–60 °C and melt flow index (MFI) of 4.03–2.01 g 10 min^−1^.

### 2.2. Synthesis of MSU-X Silica Material

MSU-X silica was synthesized at room temperature following previously reported procedures [29,30]. In a typical synthesis, 5.7 g of Triton X-100 (9 mmol) was magnetically stirred in 285 mL of distilled water until a clear solution was obtained. Then, 18.6 mL of TEOS (90 mmol) was added dropwise to the surfactant solution with stirring at room temperature. To induce the silica precipitation, 33.4 mL of a 0.05 M solution of ammonium fluoride was added. The mixture was left at room temperature during 24 h under vigorous stirring. The obtained solid was washed with water and acetone and dried at room temperature. Finally, the surfactant was easily removed by solvent extraction with ethanol at room temperature for 12 h, as confirmed by FTIR analysis.

### 2.3. TOC Incorporation into the MSU Silica Material

The incorporation of the active agent into the mesoporous silica was carried out by wet impregnation of the previously synthesized silica MSU-X with α-tocopherol (sample MSU-X-TOC (IMP)), by following the method proposed by Garguilo et al. [18] with some modifications. The desired α-tocopherol amount was dissolved in ethanol (8 mL per gram of substrate); then, MSU-X silica was added to the solution and the suspension was homogeneously mixed using an ultrasonic bath (4 series of 5 min). The wet powder was spread on a watch glass and dried at 50 °C for 24 h under stirring to favour the evaporation of ethanol thus allowing the recovery of the solid powder. The tocopherol/silica weight ratio was 0.6. This value was chosen with the aim of having the amount of α-tocopherol approximately capable of filling the total pore volume of the hosting MSU-X calculated from the adsorption isotherm.

### 2.4. Characterization of the Silica-Based Materials

The porous texture of the materials was characterized by N_2_ adsorption/desorption isotherms at 77 K. Measurements were carried out in an AUTOSORB-6 apparatus (Anton Paar GmbH, Ostfildern, Germany). Samples were previously degassed for 5 h at 373 K and 5 × 10^−5^ bars to avoid TOC degradation. Specific surface areas were calculated using the BET method in the relative pressure (P/P_0_) range of 0.05 to 0.30. Mesopore size was obtained by applying a NLDFT equilibrium model to the isotherm. Pore volume was directly read from the isotherms at a relative pressure of ca. 0.95. FTIR spectra were recorded using a Thermo Scientific Nicolet Nexus FT-IR spectrometer (Walham, MA, USA) in a wavenumber range from 4000 to 200 cm^−1^. Samples were dried before FTIR analysis to remove moisture. To determine the amount of TOC incorporated into the silica, thermogravimetric measurements (TGA) were performed using a Mettler Toledo TG/SDTA analyser (Schwarzenbach, Switzerland) under O_2_:N_2_ (1:4) atmosphere (flow rate 100 mL min^−1^) from room temperature to 900 °C at a heating rate of 10 °C min^−1^.

### 2.5. Films Preparation

Different biofilms were obtained by melt blending in a Haake Polylab QC mixer (ThermoFischer Scientific, Walham, MA, USA) at 80 °C with a mixing time of 5 min and 50 rpm of rotor speed. Prior to the mixing step, PCL and MSU-X materials with TOC were conditioned at room temperature in a desiccator for 48 h to remove the humidity and they were dried in an oven at 120 °C for 24 h in order to avoid PCL hydrolysis during processing.

Five different PCL-based formulations (Table 1) were obtained by combining PCL matrix with MSU-X silica (PCL-MSU) or with α-tocopherol (PCL-TOC); by the direct addition of MSU-X and α-tocopherol into PCL (PCL-AD); and by adding α-tocopherol impregnated in MSU-X, MSU-X-TOC (IMP) (PCL-IMP). An additional PCL neat film without any additive was also prepared and used as control (PCL). The percentage of MSU-X selected was established to obtain 2% of α-tocopherol in the final formulation.

Films were obtained by compression-moulding at 80 °C in a hot press for 8 min (Carver Inc., Model 3850, Wabash, IN, USA). The average thickness of films was around 230 ± 20 μm measured with a Digimatic Micrometer Series 293 MDC-Lite (Mitutoyo, Kyoto, Japan) at five random positions around the film.

### 2.6. Films Characterization

#### 2.6.1. Thermal Analysis

TGA tests were performed with a TGA/SDTA 851 Mettler Toledo thermal analyser. Approximately 7 mg of samples were weighed in alumina pans (70 μL) and they were heated from 25 °C to 600 °C at a heating rate of 10 °C min^−1^ under nitrogen atmosphere (flow rate 50 mL min^−1^), followed by a second step up to 900 °C at 10 °C min^−1^ under oxygen atmosphere (50 mL min^−1^) in order to ensure the complete degradation of samples.

Differential scanning calorimetry (DSC) tests were performed to determine the effect of silica and α-tocopherol addition on the thermal properties of PCL blends. Tests were conducted by using a TA Instruments DSC Q2000 (New Castle, DE, USA) under nitrogen atmosphere (flow rate 50 mL min^−1^). Three milligrams of films were introduced in aluminium pans (40 μL), and they were submitted to the following thermal program; heating from −90 °C to 200 °C at 10 °C min^−1^ (3 min hold), cooling at 10 °C min^−1^ to −90 °C (3 min hold) and heating to 200 °C at 10 °C min^−1^.

Crystallization temperature (T_c_) and enthalpy (∆*H_c_*) values were determined from the cooling scan, while glass transition temperature (T_g_), melting temperature (T_m_) and enthalpy (∆*H_m_*) parameters were obtained from the second heating scan. The degree of crystallinity (χ,%) was calculated using Equation (1):

(1) $$\chi =\frac{\Delta H_{m}^{i}}{w\times \Delta H_{m}^{0}}\times 100$$

where $\Delta H_{m}^{i}$ (J g^−1^) is the latent heat of fusion of the sample, *w* is the PCL weight fraction in the sample and $\Delta H_{m}^{0}$ (J g^−1^) is the theoretical latent heat of melting for 100% pure crystalline PCL (139 J g^−1^) [31]. All samples were analysed in triplicate and reported as the average value ± standard deviation.

The antioxidant performance of TOC and the thermo-oxidative stability of PCL-based ϕιλμσ were studied by DSC by determining the oxidation induction parameters, i.e., oxidation onset temperature (OOT (°C)) and oxidation induction time (OIT (min)). The oxidative stability can be determined by DSC by following the standardized test method ASTM E2009-08 [32]. All tests were performed in triplicate for each formulation [33]. Samples for OOT tests were heated up at 10 °C min^−1^ under pure oxygen atmosphere (50 mL min^−1^) from 25 °C until the observation of the exothermic oxidation peak. The determination of OIT values was carried out by heating samples at 100 °C min^−1^ under nitrogen (flow rate 50 mL min^−1^) to the set temperature (220 °C). After 5 min, the atmosphere was switched to pure oxygen or air (50 mL min^−1^). The heat flow was then recorded in isothermal conditions up to the detection of the exothermic peak indicating the beginning of the oxidation reaction.

#### 2.6.2. Barrier Properties

Water vapour permeability (WVP, kg m Pa^−1^ s^−1^ m^−2^) was determined gravimetrically according to UNE 53097:2002 [34] standard and it was calculated from water vapour transmission rate values (WVT, kg s^−1^ m^−2^) through mean film thickness (*e*, m) following the Equation (2):

(2) $$WPV=\frac{WVT\times e}{\Delta P}$$

where Δ*P* is the vapour pressure difference between the two sides of the films (Pa). Samples with 90 mm diameter were sealed with paraffin to the test stainless steel dishes containing anhydrous calcium chloride (dried at 200 °C for 2 h) as desiccant agent and kept in a climate chamber at 23 ± 1 °C and 50 ± 2% of relative humidity. The dishes were weighed periodically until the steady state was reached, allowing the calculation of WVT according to the standard. Three replicate samples were performed in this study.

Oxygen transmission rate (OTR, cm^3^ m^−2^ day^−1^) was determined by using an oxygen permeation analyser (Systech Instruments, model 8500, Metrotec S.A., Spain). Films (14 cm diameter circles) were clamped in the diffusion chamber at 23 °C. Pure nitrogen (99.9%) was injected into the lower half of the chamber, where an oxygen sensor was placed, while an oxygen flow (99.9%) was introduced into the upper half. Tests were performed in triplicate and mean values were expressed as oxygen transmission rate per film thickness (OTR*e).

#### 2.6.3. Optical Properties

Colour modifications on PCL films due to the incorporation of the additives were monitored by using a Konica CM-3600d COLORFLEX-DIFF2 colorimeter, HunterLab, (Reston, VA, USA). Colour values were expressed as L* (lightness), a* (red/ green) and b* (yellow/blue) coordinates in the CIELab colour space. These parameters were determined at five different locations over the film surface and average values were calculated. Total colour difference (ΔE*) was calculated according to Equation (3).

(3) $$\Delta E^{*}=\sqrt{{\left(\Delta L_{}^{*}\right)}^{2}+{\left(\Delta a_{}^{*}\right)}^{2}+{\left(\Delta b_{}^{*}\right)}^{2}}$$

where ΔL*, Δa* and Δb* are the difference between control (PCL film without any additive) and each sample values of L*, a* and b*, respectively.

### 2.7. Release Tests

The release of TOC from PCL-based films was evaluated by using ethanol 50% (*v*/*v*) as food simulant, in agreement with the European Standard EN 13130-2005 [35] and the Commission Regulation (EU) No 10/2011 on plastic materials and articles intended to come into contact with food [22]. Total immersion migration tests were performed with 12 cm^2^ films and 20 mL of the simulant (area-to-volume ratio of 6 dm^2^ L^−1^) at 40 °C in an oven (J.P. Selecta, Barcelona, Spain) for 10 days. A blank test was also carried out. Extracts were taken at different times (6, 14, 24 and 48 h and 3, 5 and 10 days), and they were stored at −4 °C before analysis. Tests were performed in triplicate.

The amount of TOC released into ethanol 50% (*v*/*v*) from the PCL-based films was determined, in triplicate, by HPLC-UV (Agilent 1260 infinity, Agilent Technologies, Santa Clara, CA, USA) at 292 nm. A BRISA LC2 C18 column (250 mm × 4.6 mm × 5 µm, Teknokroma, Barcelona, Spain) was used. The mobile phase was methanol/acetonitrile (1:1) at 1 mL min^−1^ flow rate and 20 µL of extracted samples were injected [36]. Calibration standards and working solutions of TOC were prepared in ethanol 50% (*v*/*v*).

The migration process is described by the kinetics of the diffusion of the migrant through the film and it is expressed by the diffusion coefficient, D (m^2^ s^−1^). This process is often described by the Fick’s second law in one dimension [37,38] (Equation (4)):

(4) $$\frac{\partial C}{\partial t}=D\frac{\partial^{2}C}{\partial x^{2}}$$

where C is the concentration of the active compound released by the film at time t and position x and D is the diffusion coefficient. Considering the case of a flat surface and a uniform initial distribution of concentration, the amount of diffusing substance released by the film can be estimated using Equation (5) [17]:

(5) $$\frac{M_{F,t}}{M_{F,\infty }}=1-\frac{8}{\pi^{2}}\overset{\infty }{\underset{n=0}{\sum }}\frac{1}{{\left(2n+1\right)}^{2}}\exp\left(-\frac{{\left(2n+1\right)}^{2}\pi^{2}}{4L^{2}}Dt\right)$$

where $M_{F,t}$ is the mass of the migrant in the food at a particular time t(s), $M_{F,\infty }$ is the mass of migrant in the food at equilibrium, *L* (m) is the thickness of the film and D (m^2^ s^−1^) is the diffusivity of migrant in the film.

However, when the release rate is slow and the equilibrium state is not reached at the end of the test, with the boundary condition of $\frac{M_{F,t}}{M_{P,0}}<0.6$, Equation (6) can be used to determine the diffusion coefficient [16].

(6) $$\frac{M_{F,t}}{M_{P,0}}=\frac{4}{L}{\left(\frac{Dt}{\pi }\right)}^{1/2}$$

where $M_{P,0}$ is the initial amount of migrant in the film (for a complete migration $M_{P,0}$ = $M_{F,\infty }$). Thus, the diffusion coefficient can be directly computed from the fitting of Equation (6) to experimental migration data.

### 2.8. Free Radical Scavenging Ability

The antioxidant ability of the films was analysed using the stable radical 2,2-diphenyl-1-picrylhydrazyl (DPPH) and ABTS (2,2′ azinobis (3-ethylbenzthiazoline)-6-sulfonic acid) free radical decolourization assays.

DPPH free radical-scavenging test was carried out according to a previous study with some modifications [24]. 500 μL of each extract were mixed with 2 mL of an ethanolic solution of DPPH (0.06 mM) in a capped cuvette. The mixture was shaken vigorously and kept in the dark for 60 min at room temperature before measuring its absorbance at 517 nm using a Biomate-3 UV-VIS spectrophotometer (Thermospectronic, Mobile, AL, USA). The scavenging ability of the active films was calculated as the percentage of inhibition (*I* %) following Equation (7):

(7) $$I\;\%=\frac{A_{0}-A_{sample}}{A_{0}}\times 100$$

where *A*_0_ is the absorbance of the control sample at t = 0 min and *A_sample_* is the absorbance of the tested sample after incubation for 60 min. All analyses were performed in triplicate.

ABTS free radical decolourization assay was performed according to Re et al. [39], with some modifications. The preformed radical of ABTS was generated by reacting ABTS solution (7 mM) with potassium persulphate solution (2.45 mM) in the darkness at room temperature for 16 h. The solution was then diluted with ethanol to obtain an absorbance value of 0.70 ± 0.02 at 734 nm. An aliquot of 0.150 mL of each extract was mixed with the ABTS solution. The absorbance was measured at 5 min with a Biomate-3 UV–Vis spectrophotometer using ethanol as blank sample. The radical scavenging ability of extracts against the radical ABTS was calculated as the percentage of inhibition (I %) (Equation (7)), where *A_sample_* is the absorbance of the tested sample after incubation for 5 min.

### 2.9. Antimicrobial Activity

The microorganisms used in this study were a typical Gram-negative bacteria, *Escherichia coli* (CECT 434; *E. coli*), and Gram-positive bacteria, *Staphylococcus aureus*, (*S. aureus*, CECT 239), which were supplied by the Spanish Type Culture Collection (CECT, University of Valencia, Spain). Both bacteria cultures were routinely grown overnight in Mueller Hinton Broth under aerobic conditions at 37 °C using a shaker incubator. The evaluation of the antibacterial activity of the films was carried out using overnight-diluted cell suspensions (1 × 10^4^ UFC mL^−1^) of *E. coli* or *S. aureus*. Each film sample (4.5 cm^2^) was introduced in a tube with the corresponding overnight-diluted cell suspension (per triplicate) and all tubes were incubated at 37 °C for 16 h. Then, the indirect measurement of cell numbers was performed by determining the optical density at 600 nm (OD_600_) of the blank and film samples, using a Biomate-3 UV–Vis spectrophotometer. An increase in turbidity reflects the index of bacterial growth and cell numbers (biomass) since the amount of transmitted light decreases as the cell population increases [40,41].

### 2.10. Statistical Analysis

Statistical analysis of results was performed with Statgraphics Centurion XVI statistical software. An analysis of variance (ANOVA) was carried out. Differences between average values were assessed based on the Tukey test at a confidence level of 95% (*p* < 0.05).

## 3. Results

### 3.1. Characterization of MSU-X-TOC (IMP) Materials

**Figure 1:**
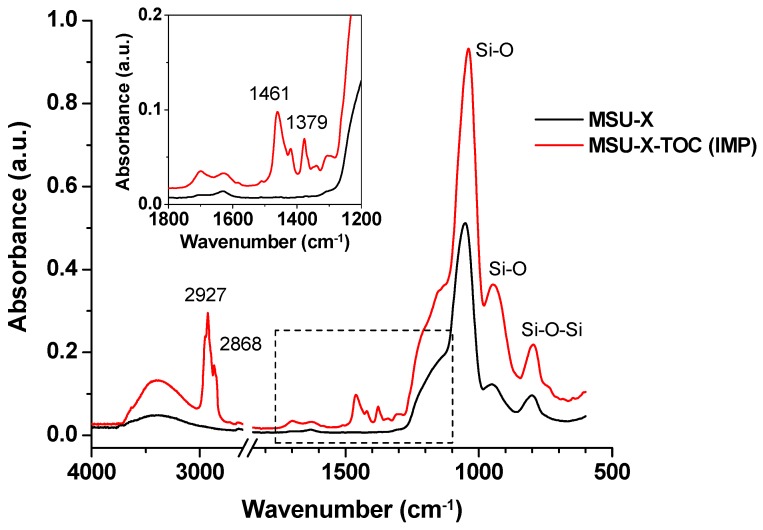
FTIR spectra of MSU-X and MSU-X-TOC (IMP) materials.

The incorporation of the active agent into the MSU-X silica, sample MSU-X-TOC (IMP), was corroborated by UV–Vis spectroscopy analysis of the filtrates, FTIR and TGA measurements. TGA analysis indicated a TOC incorporation of ca. 28 wt%, being the incorporation yield close to 100% in the latter. These values were consistent with the UV-Vis analysis performed in the filtrates. The FTIR spectra obtained for the MSU-X silica before and after the TOC loading (Figure 1) presented the characteristic silica absorption bands centred at ~1040 cm^−1^ corresponding to the vibration stretching and bending modes of the Si–O–Si bonds, along with the absorptions at 970 cm^−1^ and 3500 cm^−1^, corresponding to the vibrations of silanol groups [29,30]. The spectrum of the silica impregnated with TOC also showed the characteristic bands of the TOC centred at 3390 cm^−1^ (hydroxyl groups), 2927 and 2868 cm^−1^ (symmetric and asymmetric vibration of CH_2_ and CH_3_, respectively), 1461 cm^−1^ (phenyl skeletal), 1379 cm^−1^ (methyl symmetric bending) and 1280 cm^−1^ (methylene groups). Furthermore, as expected, the synthesized MSU-X silica presented a type IV isotherm, typical of mesoporous materials, with excellent textural properties (BET area of ca. 800 m^2^/g, pore volume of 0.8 cm^3^/g and mesopore size of ~3 nm, according to the size of the surfactant micelles). However, after α-tocopherol was trapped in the structure of the silica, the porosity of the MSU-X material was completely blocked, confirming the TOC incorporation in the MSU-X-TOC (IMP) sample.

### 3.2. Characterization of PCL-Based Films

#### 3.2.1. Thermal Properties

**Figure 2:**
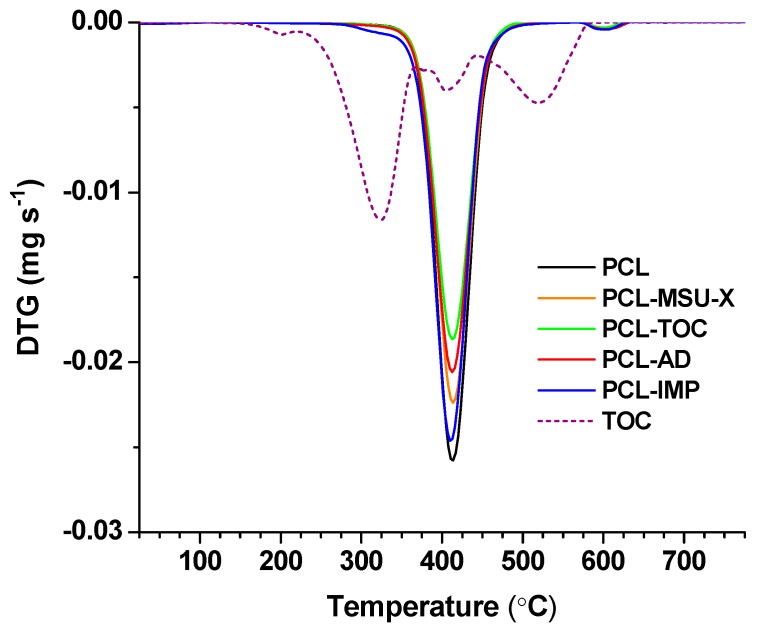
Derivative thermogravimetry (DTG) curves in nitrogen atmosphere at 10 °C min^−1^ of PCL-based films, TOC and MSU-X.

Figure 2 shows the TGA curves obtained for all the studied formulations observing one main degradation step at ~410 °C, approximately, which was related to the thermal degradation of the PCL matrix in inert atmosphere. The degradation process took place through the rupture of the polyester chains via ester pyrolysis reaction with the release of carbon dioxide, water, and carboxylic acids [42].

The initial decomposition temperature (T_ini,5%_), determined at 5% weight loss, and maximum degradation temperature values are shown in Table 2. As it can be seen, the addition of MSU-X and/or TOC or impregnated silica with TOC did not significantly affect the thermal stability of the biofilms (*p* > 0.05). Regarding the addition of α-tocopherol into the PCL matrix, no plasticizing effect was observed at the added concentration to be able to modify the thermal stability. A significant effect on the thermal stability of PCL with the addition of 10 wt% hydroxytyrosol (a similar antioxidant) due to a plasticizing effect was reported, resulting in the reduction of the initial decomposition temperature around 18 °C [42].

DSC thermal parameters (glass transition temperature, T_g_, melting temperature, T_m_, crystallization temperature, T_c_, and associated enthalpies (Δ*H_m_* and Δ*H_c_*)) of PCL-based films are reported in Table 2. For the PCL neat film, T_g_, T_m_ and T_c_ values of −64.9 ± 0.4 °C, 55.5 ± 0.1 °C and 33.8 ± 0.2 °C, respectively, were obtained. These values are consistent with those found in the literature [42,43,44]. No significant differences (*p* > 0.05) were observed for T_g_ values as a consequence of the addition of the studied additives. This effect could be probably due to the fairly high chain mobility of PCL, being the interaction between the added additives and the PCL chains not strong enough to cause a significant change in T_g_. Regarding melting temperatures, no significant differences (*p* > 0.05) between neat PCL and the rest of formulations were shown which was related to the small changes in crystallinity values obtained for the developed materials. The addition of MSU-X with or without TOC did not produce a significant modification (*p* > 0.05) of the crystallization rate. In this sense, the PCL crystalline structure was not modified by the presence of the additives and possible nucleation effects were avoided, promoting an acceptable mobility of the polymer chains. Similar results were obtained by Beltrán et al. [42] in PCL nanobiocomposite films containing hydroxytyrosol and montmorillonite, where no significant differences were observed in crystallinity values for films containing 2.5 and 5 wt% of the nanoclay.

OOT and OIT values provide relative measurements of the thermo-oxidative stability of a material at a given heating rate and oxidative environment. In addition, the determination of OOT and OIT parameters is also considered a reliable, simple and fast method for the evaluation of antioxidants efficiency, in this case TOC, to protect the polymer to oxidative degradation during processing and further use [33]. The addition of MSU-X and TOC significantly increased (*p* < 0.05) OOT (~4 °C and ~33 °C, respectively) and OIT (~19 and ~31 min, respectively) values of the polymer matrix as it can be observed in Table 3. These results denoted the high antioxidant effect of α-tocopherol when it was added into the PCL matrix. Moreover, the addition of MSU-X with α-tocopherol (PCL-AD and PCL-IMP) significantly increased (*p* < 0.05) OOT and OIT values compared to PCL and PCL-MSU-X formulations, showing more than 40 min of difference in OIT values. This behaviour depicted the effectiveness of α-tocopherol to improve the polymer stability against oxidative atmosphere when it is impregnated or directly added to MSU-X. Regarding OOT values, an increase of more than 40 °C was observed compared to neat PCL, once again demonstrating the antioxidant effect of the added additives. Similar results were obtained by other authors combining several antioxidant additives with different polymers [33,45]. PCL-MSU-X also showed a significant improvement (*p* < 0.05) in OOT and OIT values, providing an enhancement in thermal stability against oxidation by the addition of the mesoporous silica.

#### 3.2.2. Barrier Properties

One of the most important factors that should be evaluated in food packaging materials is the barrier properties against oxygen and water vapour; making necessary to control these parameters, either to obtain materials with high or low barrier properties or for applications involving the use of food packaging under controlled conditions. In general, the barrier properties of polymers are affected by several factors such as polarity, hydrogen bonding between side chains, molar mass and polydispersity, cross-linking, crystallinity and processing methodology [46].

Barrier properties to oxygen were studied by the determination of oxygen transmission rate per film thickness (e), OTR*e (Table 3). As it can be seen, higher OTR*e values were obtained for PCL-TOC films compared to neat PCL, formulations with MSU-X (PCL-MSU-X), and MSU-X with α-tocopherol (PCL-IMP and PCL-AD). This behaviour could be due to the modification of the polymer matrix structure in the presence of TOC, consequently reducing the resistance of films to oxygen diffusion through them [42]. The chemical interaction between polymer chains and the additive molecules produces an increase in the free volume [47]. In the presence of silica, a decreasing trend in OTR*e values was observed although it was not significantly different (*p* > 0.05) compared to PCL. These results could suggest that the incorporation of the mesoporous silica with or without TOC could promote the intercalation through the polymer matrix or the interaction between the polymer chains and the silica, rendering more competitive materials for oxygen-sensitive products.

The results obtained for water vapour permeability (WVP) in PCL-based films are summarized in Table 3. No significant differences (*p* > 0.05) between formulations were observed by comparing WVP values after the incorporation of α-tocopherol, despite the hydrophobic nature of this antioxidant. These results are in agreement with those obtained by DSC analysis, not showing a significant plasticizing effect probably due to the low content of the active compound present into the PCL matrix. Other authors have reported a plasticizing effect with increase in free volume and chain mobility induced by the addition of α-tocopherol in LDPE [17] films or carvacrol in PLA [48] and PLA-PHB [49] formulations, at higher additives concentration. These results suggest that there should be a balance between the silica and TOC added contents to significantly modify the barrier properties of the PCL matrix, according to the final application.

#### 3.2.3. Optical Properties

Colour is an important factor to be considered in food packaging applications as it could influence the consumer acceptance and commercial success of the final product. In the present study, the chromatic parameters (L*, a* and b*) and the total colour difference (Δ*E*) were evaluated to study the effect of the added additives on the PCL-based films colour (Table 4). Colour can be defined in terms of the CIELab colour space, where the individual values: a* and b* represent the colour features of PCL-based films, while L* is the lightness and refers to the relation between reflected and absorbed light. A uniform distribution of colour was observed in all films due to the high homogeneity distribution of the additives within the polymer matrix. Significant differences (*p* < 0.05) in a* and b* values between neat PCL and PCL-based films were observed (Table 4). In particular, the direct incorporation of TOC (PCL-TOC, PCL-AD) or its impregnation into the silica (PCL-IMP) provided a yellowish-reddish colour, producing higher values of ΔE. These differences could be attributed to the intrinsic colour of the additives used, as reported by other authors in PCL films containing hydroxytyrosol [42]. Moreover, Byun et al. [24] found that the presence of α-tocopherol and resveratrol in PLA films contributed to strength the final colour.

### 3.3. Release Tests

**Figure 3:**
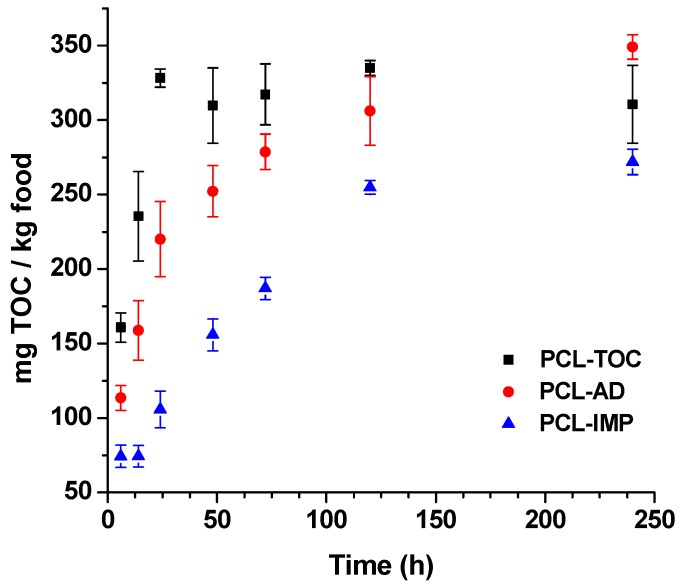
Release of α-tocopherol from PCL-TOC, PCL-AD and PCL-IMP films into ethanol 50% (v/v) over 10 days (mg per kg of food).

A release study of α-tocopherol was carried out by using ethanol 50% (v/v) as food simulant to evaluate the functionality of the developed materials for food packaging applications. The release was monitored at different times and the amount of α-tocopherol was measured by HPLC-UV analysis. The results obtained are depicted in Figure 3. Highest release values were observed for PCL-TOC formulation, with no silica into the polymer matrix, reaching a maximum value of 328.3 ± 6.1 mg α-tocopherol per kg simulant within the first 24 h of exposure into the food simulant. After 24 h, no significant differences (*p* > 0.05) were observed in the release concentration with time, reaching this formulation its steady state. Formulations containing α-tocopherol and silica (PCL-AD and PCL-IMP) showed lower α-tocopherol diffusivity during the study test.

In general terms, the fastest TOC release was obtained during the first 24 h, showing a linear increase trend for the three studied formulations. However, PCL-TOC showed the biggest slope and highest migration values whereas PCL-IMP showed the lowest release values during the study. This behaviour observed in PCL-IMP films could be indicative of the interactions established between α-tocopherol and silica as a result of the impregnation process that occupies all the silica porosity, retaining α-tocopherol to a greater extent in the polymer matrix. In the case of PCL-AD, some interactions may occur, but the encapsulation of the active additive in the silica is less favourable. These interactions and the affinity between both silica and α-tocopherol resulted in the reduction of the release of α-tocopherol from the PCL matrix into the food simulant.

These results demonstrate the possibility of controlling the release of natural agents through the incorporation of silica impregnated with natural additives, such as α-tocopherol, with direct application for the design of novel active films. Several authors have studied the release of α-tocopherol in different food simulants, confirming its effectiveness as an active agent to increase the shelf-life of food. A similar behaviour was found for Barbosa-Pereira et al. [50] when developing antioxidant active films containing α-tocopherols to be used as active packaging systems in the food industry to extend the shelf-life of salmon. Gargiulo et al. [18] observed that α-tocopherol was released more slowly from LDPE films loaded with functionalized SBA-15 mesoporous silica compared to the direct incorporation of the additive.

**Figure 4:**
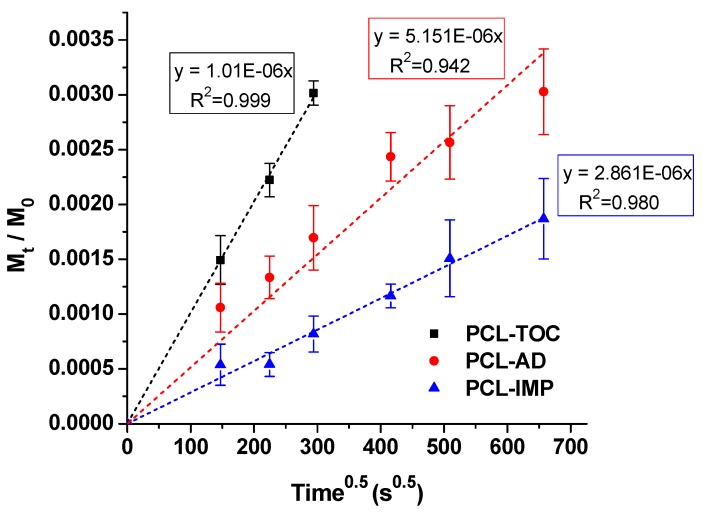
Mass fraction versus the square root of time and curves fitted for α-tocopherol released from PCL-based films into 50% ethanol. Dotted curves represent the migration model fitted to the corresponding experimental data points.

The determination of the diffusion coefficients of α-tocopherol in these systems is an important issue for the development of active packaging materials. The assessment of the release mechanism was performed by using an approach based on the Fick’s second law. Based on Equation (6), the validity of this approach is restricted due to the fact that PCL-AD and PCL-IMP films did not reach the steady state after five days. Therefore, the determination of the migration kinetics associated to this process was determined by using the approximation for short times suggested by Crank [37]. This approach assumes a linear variation of the concentration of an active agent versus the square root of time, where the slope gives information about the diffusion coefficient of α-tocopherol in the PCL matrix, according to Equation (6) discussed above. Figure 4 shows the fitting of Equation (6) to the experimental data and the computed fitting parameters. The fittings follow the same trend than those observed in Figure 3, being the slope for PCL-TOC the highest, while the smallest value was obtained for the PCL-IMP formulation, where MSU-X was impregnated with α-tocopherol. The calculated diffusion coefficients for α-tocopherol desorption from PCL-TOC, PCL-AD and PCL-IMP were 30.2 × (±0.9) × 10^−15^ cm^2^ s^−1^, 8.0 × (±0.7) × 10^−15^ cm^2^ s^−1^ and 3.5 × (±0.3) × 10^−15^ cm^2^ s^−1^, respectively. These values showed a slower release of α-tocopherol from formulations containing silica compared to PCL-TOC. Therefore, it can be concluded that the transport mechanism through the polymer matrix will be different in the presence or absence of silica delaying the α-tocopherol release when MSU-X is present, with direct application as a potential food packaging material.

Manzanares-López et al. [25] reported the release of α-tocopherol from PLA films at different times and temperatures, and they obtained a diffusion coefficient value in pure ethanol of 38.00 ± 2.90 × 10^−11^ cm^2^ s^−1^ at 43 °C. This value is 4 and 5 orders of magnitude higher than those obtained in this study for PCL-TOC and PCL-silica-tocopherol systems, respectively. The main difference between both studies, besides the polymeric matrix, is the simulant used for performing the migration study. This behaviour could be explained by the higher affinity of pure ethanol for α-tocopherol compared to ethanol 50% (*v*/*v*) (simulant D1 in EN 13130-2005 standard). The affinity between the food simulant and the active compound and its effect in the final release was evaluated by Castro López et al. [51] in a PP matrix with α-tocopherol at 40 °C. They found that the diffusion coefficient value increased from 2.1 × 10^−15^ cm^2^ s^−1^ using simulant A (ethanol 10%, *v*/*v*) up to 3.1 × 10^−12^ cm^2^ s^−1^ when simulant D1 was employed. The authors concluded that the diffusion coefficient not only depends on the active agent being released, but also on the polymer matrix, temperature and food simulant used.

The differences found between the diffusion coefficient values for formulations containing silica (PLC-AD and PCL-IMP), showing a delay in the α-tocopherol release with time, and without silica (PCL-TOC) could be related to the chemical interactions present between the three components of the system. The hydroxyl group present in α-tocopherol could interact with the carbonyl group of PCL, retaining the active compound and thus reducing its capacity to diffuse into the simulant. Furthermore, when α-tocopherol is incorporated into silica MSU-X, hydrogen bonds are established between the hydroxyl groups, thus decreasing the diffusion rate of α-tocopherol, in agreement with previous studies that proved the efficiency of functionalized SBA-15 mesoporous silica to delay the release of α-tocopherol adsorbed in the silica and incorporated into LDPE films [18]. Migration tests were performed at 25 °C using ethanol 96% (v/v) as food simulant and they found that the diffusivity of α-tocopherol from films containing functionalized SBA-15 decreased by 60% compared to the films with free α-tocopherol. Sun et al. [17] also demonstrated a decrease by 53% in the diffusivity of α-tocopherol loaded with MCM-41 mesoporous silica through LDPE films. Similarly, Li et al. [16] observed a synergistic release of α-tocopherol and quercetin due to the controlled release effect of MCM-41, obtaining a diffusion coefficient of 7.71  ×  10^−15^ cm^2^ s^−1^.

### 3.4. Free Radical Scavenging Ability

**Figure 5:**
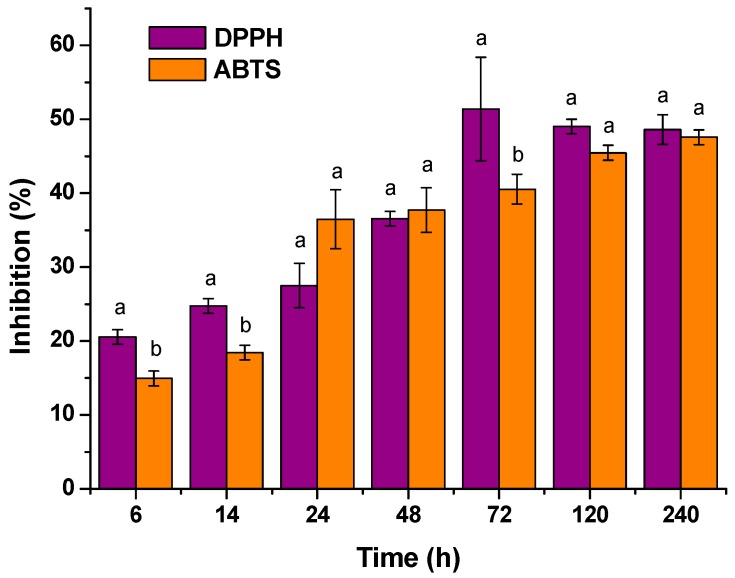
Comparison between DPPH and ABTS methods for PCL-TOC film (mean ± SD; *n* = 3). Different letters (a, b) at the same time indicate statistically different values between the studied methods (*p* < 0.05).

The importance of antioxidant methods lies in the need to know the ability of the obtained active materials to protect the food from oxidative processes that could shorten the shelf-life of food, causing serious organoleptic deterioration and therefore consumer rejection. The antioxidant capacity of the extracts obtained from the release tests of PCL-TOC formulation were compared by following DPPH and ABTS methods. Results were expressed as percentage of inhibition (I %) (Figure 5). In general terms, no significant differences (*p* > 0.05) between inhibition values after 24 h of study were found, suggesting that, in both methods, the free radical reagent may be neutralized either by direct reduction via electron transfer or by radical quenching via H atom transfer [52].

**Figure 6:**
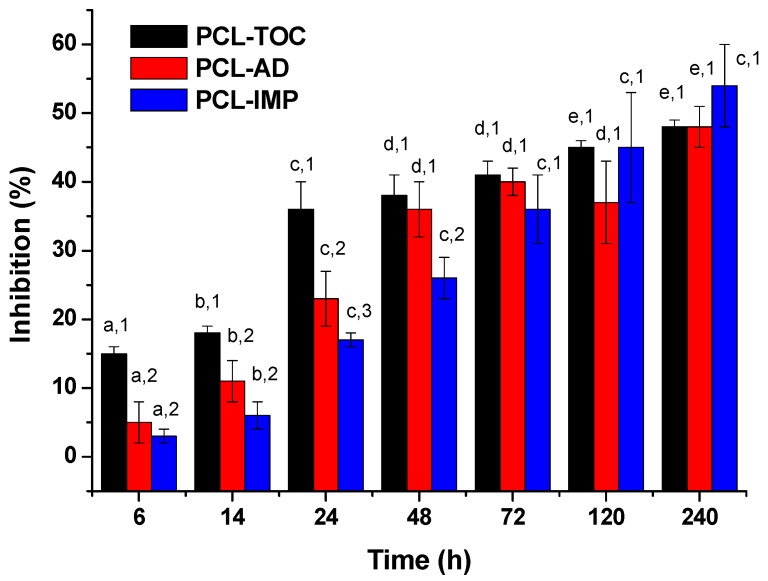
ABTS results of migration extracts determined by ATBS method (mean ± SD; *n* = 3). Different numbers (1, 2) at the same time indicate statistically different values between formulations (*p* < 0.05) and different letters (a, b, c, d, e) at the same formulation indicate statistically different values between times (*p* < 0.05).

Figure 6 shows the percentage of inhibition obtained using the ABTS method for PCL-TOC, PCL-AD and PCL-IMP films. At short initial times, the percentage of inhibition obtained for PCL-TOC at 6 h was the highest (15 ± 1%) compared to those obtained for PCL-AD and PCL-IMP (5 ± 3% and 3 ± 1%, respectively). In the first 24 h, the antioxidant activity was also significantly higher (*p* < 0.05) for PCL-TOC formulation. These results are in agreement with the higher amount of α-tocopherol released and diffusion coefficient values obtained in the release tests for PCL-TOC. No significant differences (*p* > 0.05) were observed between inhibition values for PCL-AD and PCL-IMP formulations. However, after 48 h, a significant increase in the antioxidant capacity of PCL-AD sample was observed, reaching a similar antioxidant capacity than that of PCL-TOC. Regarding PCL-IMP films, the interaction between the matrix, silica and active additive seems to be greater than the affinity for the simulant, retaining in a higher extend the active additive and slowing its release, therefore affecting the antioxidant capacity of the final formulation. However, as the study was progressing and the release reached a stationary state, the equilibrium between the matrix and the simulant were more established and inhibition values of all the studied formulations were similar, showing no significant differences between them at final times (*p* > 0.05). The relationship between the diffusion coefficient and antioxidant capacity of active compounds has been studied by different authors [19,53,54,55]. They concluded that samples which exhibit higher diffusion coefficients generally show higher antioxidant capacity before reaching the steady state.

### 3.5. Antimicrobial Activity

The antibacterial activity of the active PCL-based films was evaluated against two representative food-borne bacteria: Gram-positive (*S. aureus*) and Gram-negative bacteria (*E. coli*) by using the optical density method. The results of the antibacterial tests for all formulations are shown in Table 5. Significant differences in cell viability (*p* < 0.05) for both bacterial strains were observed for all formulations compared to neat PCL at the tested experimental conditions, indicating that α-tocopherol was able to provide antibacterial capacity when it was incorporated into the PCL matrix, where α-tocopherol can diffuse and inhibit the bacterial growth. However, in the case of PCL-IMP formulation, no significant differences (*p* > 0.05) were observed in the optical density values for *E. coli* compared to neat PCL. This result can be explained by the lower coefficient of diffusion previously determined for this formulation, as the concentration of α-tocopherol released after 16 h of incubation into the culture medium of *E. coli* might be not enough to produce a significant inhibition growth. Similar results were reported by other authors for different polymer matrices and active compounds [33]. Al-Salih et al. [56] studied the antibacterial effect of vitamin E against several Gram-positive and -negative bacteria. They concluded that Gram-negative bacteria showed higher resistance towards antibacterial substances, such as α-tocopherol, that can be related to the lipo-polysaccharides present in their outer membrane. Thus, higher amounts of α-tocopherol should be required in Gram-negative bacteria to obtain the same antibacterial effect that in Gram-positive. In this sense, Gram-positive bacteria have cell membrane covered by a cell wall made up of 30–40 layers of peptidoglycans; whereas Gram-negative bacteria are composed of an outer membrane wherein phospholipids (LPS) and proteins are held together by electrostatic interactions with divalent metal ions, 1–2 layers of peptidoglycans (cell wall) and a cell membrane of lipid bilayer [57]. In other study, Tintino et al. [58] demonstrated the effectiveness of α-tocopherol by inhibiting the growth of *S. aureus* strains. Therefore, it can be concluded that the incorporation of 2 wt% of α-tocopherol either by direct addition or impregnated into MSU-X to PCL matrices showed high potential for the development of antibacterial films.

## 4. Conclusions

In this work, the effect of the addition of α-tocopherol (TOC) on MSU-X mesoporous silica for the development of PCL-based active films was studied. The presence of MSU-X proved to exert a great influence over the antioxidant and antimicrobial activity of TOC-containing PCL films. Both PCL-AD (direct addition of TOC and MSU-X) and PCL-IMP (TOC impregnated into MSU-X silica) films showed good thermal stability according to TGA results and no significant changes in oxygen and water vapour barrier properties. The increase in oxidation onset parameters values (OOT, OIT) obtained for these formulations indicated the effectiveness of the added mesoporous silica and antioxidant TOC to protect the final material from thermal oxidation and degradation favouring its processing at high temperatures and further use. PCL-IMP showed a slower antioxidant release in ethanol 50% (v/v), when compared to the other PCL-based films studied containing free α-tocopherol without MSU-X (PCL-TOC) and with the silica (PCL-AD). Indeed, the antioxidant diffusivity of PCL-IMP films decreased by 10 times with respect to films containing free α-tocopherol. The results obtained for antioxidant and antimicrobial tests showed similar trends for PCL-IMP and PCL-AD films compared to PCL-TOC film based on kinetic release, exhibiting higher antibacterial activity against Gram-positive strains (*S. aureus*) compared to Gram-negative bacteria (*E. coli*). The impregnation of α-tocopherol in MSU-X silica and further addition to the PCL matrix showed the most promising results obtaining the lower amount of α-tocopherol released into the food simulant at short times but attractive functional properties for antimicrobial/antioxidant food packaging applications. The obtained results offer new insight into the use of mesoporous silicas for the development of controlled-release systems for active packaging. To complete these investigations for further use at large scale, further tests are needed to be carried out such as the evaluation of mechanical performance of the developed materials.

## Figures and Tables

**Table 1:** Different formulations obtained in this work and their codification.

Formulation	Component Content (wt%)			
	Code	MSU-X	α-Tocopherol	Impregnated MSU-X
PCL (neat film)	PCL	0	0	0
PCL + MSU-X	PCL-MSU	4.9	0	0
PCL + α-tocopherol	PCL-TOC	0	2	0
PCL + MSU-X + TOC	PCL-AD	4.9	2	0
PCL + MSU-X-TOC (IMP)	PCL-IMP	0	0	6.9

**Table 2:** Thermal parameters obtained by DSC and TGA (N_2_ atmosphere) in PCL-based films.

Samples	TGA		DSC					
			Second Heating Scan			Cooling Scan		
	T_ini,5%_ (°C)	T_max_ (°C)	T_g_ (°C)	T_m_ (°C)	ΔH_m_ (J/g)	T_c_ (°C)	ΔH_c_ (J/g)	χ (%)
PCL	378 ± 1 ^a^	412 ± 2 ^a^	−64.9 ± 0.4 ^a^	55.5 ± 0.1 ^a^	51.4 ± 1.6 ^a^	33.8 ± 0.2 ^a^	54.7 ± 1.7 ^a^	37 ± 1 ^a^
PCL-MSU-X	377 ± 2 ^a^	411 ± 1 ^a^	−63.7 ± 0.6 ^a^	55.0 ± 0.2 ^a^	43.0 ± 3.0 ^b^	30.7 ± 1.8 ^b^	46.9 ± 0.7 ^b^	32 ± 2 ^b^
PCL-TOC	380 ± 2 ^a^	410 ± 2 ^a^	−64.0 ± 3.0 ^a^	55.2 ± 0.2 ^a^	48.0 ± 6.0 ^b^	32.7 ± 0.3 ^b^	47.0 ± 0.5 ^b^	35 ± 4 ^a^
PCL-AD	377 ± 4 ^a^	410 ± 4 ^a^	−62.5 ± 0.7 ^a^	55.3 ± 0.2 ^a^	43.9 ± 1.8 ^b^	33.0 ± 0.4 ^ab^	43.0 ± 4.0 ^c^	34 ± 1 ^b^
PCL-IMP	372 ± 7 ^a^	409 ± 3 ^a^	−63.8 ± 0.7 ^a^	55.0 ± 0.2 ^a^	44.5 ± 1.8 ^b^	32.3 ± 0.1 ^ac^	42.7 ± 1.9 ^c^	34 ± 1 ^b^

Different superscripts (a, b, c) within the same column indicate statistically different values (*p* < 0.05). Mean ± SD; *n* = 3.

**Table 3:** OOT and OIT (DSC, O_2_ atmosphere); WVP and OTR*e parameters in PCL-based films.

Sample	OOT (°C)	OIT (min)	WVP × 10^14^ (kg m Pa^−1^ s^−1^ m^−2^)	OTR*e (cm^3^ mm m^2^ Day)
PCL	237 ± 2 ^a^	9 ± 1 ^a^	2.35 ± 0.13 ^a^	76 ± 15 ^a^
PCL-MSU-X	241 ± 2 ^b^	28 ± 9 ^b^	3.31 ± 0.61 ^a^	60 ± 15 ^a^
PCL-TOC	270 ± 2 ^c^	40 ± 4 ^b^	2.22 ± 0.03 ^a^	87 ± 2 ^a^
PCL-AD	279 ± 1 ^d^	52 ± 7 ^c^	2.23 ± 0.10 ^a^	66 ± 4 ^a^
PCL-IMP	283 ± 2 ^d^	63 ± 9 ^c^	2.79 ± 0.93 ^a^	63 ± 11 ^a^

Different superscripts (a, b, c, d) within the same column indicate statistically different values (*p* < 0.05). Mean ± SD; *n* = 3.

**Table 4:** CIELab colour parameters obtained in PCL-based films.

Samples	L*	a*	b*	Δ*E*
PCL	57.61 ± 1.44 ^ab^	−1.24 ± 0.02 ^a^	−4.20 ± 0.15 ^a^	-
PCL-MSU-X	58.33 ± 0.84 ^ab^	−1.29 ± 0.01 ^ab^	−3.51 ± 0.06 ^b^	1.16 ± 0.42 ^a^
PCL-TOC	55.93 ± 0.95 ^a^	−1.33 ± 0.06 ^b^	−3.16 ± 0.10 ^c^	2.03 ± 0.78 ^ab^
PCL-AD	59.53 ± 0.46 ^b^	−1.44 ± 0.01 ^c^	−2.46 ± 0.11 ^d^	2.61 ± 0.19 ^b^
PCL-IMP	55.66 ± 1.45 ^a^	−1.50 ± 0.07 ^c^	−2.79 ± 0.23 ^e^	2.57 ± 0.87 ^b^

Different superscripts (a, b, c, d, e) within the same column indicate statistically different values (*p* < 0.05). Mean ± SD; *n* = 3.

**Table 5:** Antimicrobial activity of PCL-based films performed by optical density method (OD_600_) after 16 h of incubation against *E. coli* and *S. aureus*.

Bacteria	Optical Density (OD_600_) per g of Sample			
	PCL	PCL-TOC	PCL-AD	PCL-IMP
*E. coli*	1.15 ± 0.19 ^a^	0.71 ± 0.15 ^b^	0.74 ± 0.06 ^b^	1.01 ± 0.08 ^ab^
*S. aureus*	0.90 ± 0.17 ^a^	0.33 ± 0.17 ^b^	0.46 ± 0.06 ^b^	0.47 ± 0.11 ^b^

Different superscripts (a, b) within the same row indicate statistically different values (*p* < 0.05). Mean ± SD; *n* = 3.
